# The ability of cancer-specific and generic preference-based instruments to discriminate across clinical and self-reported measures of cancer severities

**DOI:** 10.1186/1477-7525-9-106

**Published:** 2011-11-28

**Authors:** Paulos Teckle, Stuart Peacock, Helen McTaggart-Cowan, Kim van der Hoek, Stephen Chia, Barb Melosky, Karen Gelmon

**Affiliations:** 1 Canadian Centre for Applied Research in Cancer Control (ARCC), Vancouver, BC, Canada; 2 Cancer Control Research, British Columbia Cancer Agency, Vancouver, BC, Canada; 3 School of Population and Public Health, University of British Columbia, Vancouver, BC, Canada; 4 Medical Oncology, British Columbia Cancer Agency, Vancouver, BC, Canada

**Keywords:** Quality of life, cancer-specific instruments, generic instruments, external validity: responsiveness, disease severity, utilities

## Abstract

**Objective:**

To evaluate the validity of cancer-specific and generic preference-based instruments to discriminate across different measures of cancer severities.

**Methods:**

Patients with breast (n = 66), colorectal (n = 57), and lung (n = 61) cancer completed the EORTC QLQ-C30 and the FACT-G, as well as three generic instruments: the EQ-5D, the SF-6D, and the HUI2/3. Disease severity was quantified using cancer stage, Eastern Cooperative Oncology Group Performance Status (ECOG-PS) score, and self-reported health status. Comparative analyses confirmed the multi-dimensional conceptualization of the instruments in terms of construct and convergent validity.

**Results:**

In general, the instruments were able to discriminate across severity measures. The instruments demonstrated moderate to strong correlation with each other (r = 0.37-0.73). Not all of the measures could discriminate between different groups of disease severity: the EQ-5D and SF-6D were less discriminative than the HUI2/3 and the cancer-specific instruments.

**Conclusion:**

The cancer-specific and generic preference-based instruments demonstrated to be valid in discriminating across levels of ECOG-PS scores and self-reported health states. However, the usefulness of the generic instruments may be limited if they are not able to detect small changes in health status within cancer patients. This raises concerns regarding the appropriateness of these instruments when comparing different cancer treatments within an economic evaluation framework.

## Introduction

Cancer is the leading cause of death in many developed countries. In Canada, the latest statistics confirm that cancer-related mortality is now higher than mortality from circulatory diseases [1]. As such, the demand for effective and efficacious treatments is rising. New cancer therapies are being developed, and approved, with the aim of improving a patient's prognosis [1,2]. These treatments, however, often have a detrimental effect on the patient's quality of life (QOL). As the therapies are administered in accordance to the patient's severity level, it is important to have a valid QOL instrument which can discriminate across all levels of disease severity. This is of importance to oncologists as defining prognostic determinants may aid in the stratification of randomization on known prognostic factors in clinical trials and in therapeutic decision-making in routine practice while maintaining a high level of QOL for the patient.

QOL can be evaluated using either disease-specific or generic preference-based instruments. Disease-specific instruments have the capacity to detect minimal changes in a specified health condition [3]. In oncology, the two most widely used instruments to assess QOL are the European Organization of Research and Treatment in Cancer (EORTC) Quality of Life Core 30 (QLQ-C30) and the Functional Assessment of Cancer Therapy - General (FACT-G) [4,5]. While the advantage of using cancer-specific instruments is their capacity to detect minimal changes in a disease, these instruments are not suitable for comparisons across different disease states. As a result, the use of generic preference-based instruments is a better option. The advantage of generic instruments is that they integrate different aspects of a health state into a single index anchored by a value of one for perfect health and zero for dead. This value can be combined with the length of time in that health state to generate a quality-adjusted life year (QALY), a metric used in economic evaluations [6]. The most commonly used generic preference-based instruments are the EuroQol 5D (EQ-5D), the Short Form 6D (SF-6D), and the Health Utilities Index (HUI) [7-9].

The use of generic preference-based instruments can be incorporated into a general health policy model to compare the efficiency of different programs or treatment strategies [6]. This provides a framework for decisions concerning the adoption of new treatments within a publicly-funded health care system. However, generic instruments typically cover dimensions of health such as mobility, pain, activity limitation, and anxiety or depression; these dimensions may not be sensitive, or relevant, to treatment effects for the health condition under investigation [3]. This may be due, in part, as to why many cancer trials do not include generic preference-based instruments; instead, focusing on cancer-specific instruments to evaluate outcomes of patients.

Currently, the validity of QOL instruments to discriminate between different levels of cancer severity has not yet been adequately evaluated. Therefore, the objective of this study is to evaluate the validity of cancer-specific and generic preference-based instruments in terms of their ability to distinguish between different measures of cancer severity. Disease severity was measured in three ways: cancer stage; Eastern Cooperative Oncology Group Performance Status (ECOG-PS) score; and patient-reported general health status.

## Methods

### Study Participants

To participate in the study, patients had the following criteria: be diagnosed with either breast, colorectal, or lung cancer; be 18 years and older; be able to speak and read English; have a life expectancy of at least six months; be without cognitive impairments; and have plans to return to an appointment with a medical oncologist. Breast, colorectal, and lung cancer were chosen as they are among the most common cancers diagnosed in British Columbia and Canada [1,2]. Recruitment and informed consent were undertaken by a medical oncologist. Consented patients were given the questionnaires to complete at a subsequent outpatient visit at the Vancouver Cancer Clinic.

To complete the study, patients had two options available to them. The instruments could be completed face-to-face with a trained research assistant at the patient's appointment. Alternatively, the patients could take the instruments home and post the completed forms in a provided pre-paid envelope. For both options, researchers were available to answer questions if needed. The order of the QOL instruments was randomized for each participant. The study protocol was approved by the Research Ethics Board of the British Columbia Cancer Agency.

The study was piloted with 66 cancer patients at the Vancouver Cancer Clinic. The objectives of this pilot study were, not only to determine the practicality of collecting five QOL measures in terms of administration and respondent burden, but also to estimate the median, mean and standard deviation (SD) of the different QOL measures; the latter provided the estimates to calculate a sample size for the main study.

Based on results from the pilot study and other preference-based instruments, a difference of 0.05 in mean utility measures of health states is considered important and meaningful [10,11]. Using 80% power to detect a difference in mean health state of 0.05 between different severity groups and assuming that the common SD is 0.10 using an independent t-test at the 5% significant level indicates that a minimum sample size of 32 in each group is needed. The mean (SD) for the EQ-5D, SF-6D, HUI-2, and HUI-3 were 0.81 (0.17), 0.71 (0.11), 0.83 (0.13), and 0.76 (0.23), respectively. To compare differences in mean health scores, we will need a sample of 62, 38, 53, and 167 respondents for EQ-5D, SF-6D, HUI-2, and HUI-3 respectively. We therefore collected data from 182 patients.

### Data Collection

#### Socio-Demographic and Clinical Information

Data pertaining to the patients' socio-demographic information were obtained using a self-administered questionnaire. The patient's cancer status, in terms of disease stage and ECOG-PS score, was extracted from their medical records. The stage of cancer is typically classified from stage 1 to stage 4; the higher the stage, the more aggressive and fast growing the cancer. The ECOG-PS is a single item rating of the degree to which patients are able to participate in typical activities without a need for rest. The scale, ranging from zero (fully active) to five (dead), assesses disease progression and its impact on the patient's daily living abilities [12]. The ECOG-PS was chosen because it is a powerful predictor of QOL and an important concern in cancer care [13]. In addition to the clinical measures, the patient provided a self-reported health status; this was measured on a seven-point response scale, ranging from excellent to fair to extremely poor.

#### Cancer-Specific Instruments

For this study, two cancer-specific instruments were used: the EORTC QLQ-C30 and the FACT-G [4,5]. The QLQ-C30 dominates cancer clinical trials in Canada and Europe, while the FACT-G is more widely used in the USA [14]. The QLQ-C30 and the FACT-G contain different items even though they cover the same scales or dimensions, respectively.

The QLQ-C30 is a 30-item questionnaire composed of multi-item scales and single items to reflect the multidimensional nature of QOL in cancer [4]. It incorporates five functional scales (i.e., physical, role, cognitive, emotional, and social), three symptom scales (i.e., fatigue, pain, and nausea and vomiting), and seven single items (i.e., dyspnea, appetite loss, sleep disturbance, constipation, and diarrhea); these are measured on a four-point response scale. The instrument also contains an item assessing the perceived financial impact of the disease and treatment and two seven-point response scales pertaining to global health and QOL. While the QLQ-C30 does not yield an overall score, a global health status score was created from the patients' responses to the two response scales relating to global health and QOL [15,16].

The fourth version of the FACT-G consists of 27 items covering four dimensions of well-being: physical, social/family, emotional, and functional [5]. Items within these dimensions are evaluated on a five-point response scale. Using the instrument developer's algorithms, an overall score and four dimension scores can be generated with higher scores reflecting better QOL. Reliability and validity, including responsiveness, of the instrument have been well documented in cancer trials and clinical settings [17-19].

#### Generic Instruments

The EQ-5D questionnaire consists of a general health descriptive system based on five items and a 100-point visual analogue scale. The five items cover mobility, self care, usual activities, pain/discomfort, and anxiety/depression with three levels per item (i.e., no problem, some problems, and extreme problems). The instrument describes 243 possible health states, which are assigned utilities based on country-specific algorithms developed by the EuroQol group. The most widely used utility algorithm was based on a time trade-off (TTO) survey of 2997 UK respondents [9]. Recently, Shaw et al [20] developed a utility algorithm based on TTO responses from 4048 US residents. In the absence of a Canadian algorithm, this was used to calculate EQ-5D utilities for this study.

The SF-6D was constructed from a sample of 11 items selected from the Short Form 36 (SF-36). These items were valued by a representative sample of the UK general population using the standard gamble (SG) [21,22]. This is a six-dimensional health state classification system with each dimension having four to six levels; therefore, 18,000 health states are described. In place of a Canadian utility algorithm, the UK population tariff was used.

A version of the HUI instrument that combines features of the HUI mark 2 (HUI-2) and HUI mark 3 (HUI-3) was used in this study. The HUI2/3 contains 15 items that focuses on aspects of vision, hearing, speech, emotion, pain, mobility, dexterity, cognition, and self-care; each item was defined by four to six levels. Using the responses on the HUI2/3, two different utilities were estimated using an algorithm developed from random samples of the Canadian population: one for the HUI-2 and one for the HUI-3 [7].

### Data Analysis

Descriptive statistics were used to characterize the sample in terms of age, sex, marital status, ethnicity, employment status, education level, and annual income. In addition to these socio-demographic variables, the patients were characterized by disease severity. Continuous variables are presented as means and SDs while categorical variables are presented as the proportion of the sample within each group. The QOL scores of the investigated instruments are reported as Tukey's values.

Before testing the ability of the instruments to discriminate across disease-severity measures, the psychometric properties of the cancer-specific instruments in terms of internal consistency and construct validity were examined. Internal consistency was evaluated using Cronbach's alpha coefficient [4,5] and convergent validity using correlation coefficients [23]. Construct validity assesses whether scales from different instruments, measuring similar dimensions of QOL, are strongly correlated with each other. Both parametric and non-parametric (Pearson and Spearman) correlation coefficients were calculated; however, as the results were statistically similar, results from the Pearson's correlation coefficients are reported. A coefficient of greater than 0.5 or less than -0.5 indicates a strong correlation between instruments, 0.30 to 0.49 or -0.49 to -0.30 a moderate correlation, and values between 0.30 to -0.30 a weak correlation [24]. We also compared the correlation between the general health scores from the cancer-specific instruments and the utility indices from the preference-based instruments. A Bonferroni correction was applied to counteract the problem of multiple comparisons [25].

The external validity of each instrument was assessed based on its ability to discriminate between different cancer severity as represented by cancer stage, ECOG-PS score, and self-reported health status. This was determined using the instruments' global scores. Patients with the greatest disease severity (i.e., cancer stage 4, ECOG-PS score 3, and very poor self-reported health) were hypothesized to have lower QOL scores across all instruments. One-way analysis of variance (ANOVA) evaluated the differences among QOL scores when stratified by the aforementioned variables of disease severity.

The effect size, the standardized mean difference between two groups on a measured outcome, was also calculated. Each of the disease severity variables were sub-divided into two meaningful groups of sufficient size: cancer stages 1-2 *versus *cancer stages 3-4; ECOG-PS 0 *versus *ECOG-PS 1-3; self-reported health status excellent-good *versus *self-reported health status fair-very poor. Stage 3 and stage 4 were grouped together because the aim was to compare late stage disease with those patients in stages 1 and 2 (early disease stages). The reason why the ECOG-PS 1-3 categories are collapsed together is due to the small number of patients in PS 3 (n = 5). The decision was made to differentiate patients who reported "no problem" with their daily lives (PS 0) and those who reported some level of problems (PS 1-3). While it might appear slightly counterintuitive to combine the fair self-rating with poor and very poor, the decision was based purely on the number of patients belonging in the two groups: excellent-good (n = 116) and fair-very poor (n = 63); including the 'fair' respondents with the 'excellent-good' would result in only 27 patients in the 'poor-very poor' group.

An effect size of one indicates a clinically meaningful change in magnitude equivalent to one standard deviation (SD). The absolute value of effect sizes (*d*) can be categorized as small (*d *= 0.2-0.5), medium (*d *= 0.5-0.8), or large (*d *> 0.8) [26]. By comparing the effect sizes across the different cancer-specific and generic preference-based instruments, their discriminative abilities can be assessed [26,27]. All analyses were performed using the STATA statistical software package, version 11.1[28].

## Results

### Patient Characteristics

One hundred and ninety five patients were approached to participate in the study. All gave consent to participate in the study. The questionnaires were completed by 184 patients; a high response rate of 94% was achieved. The average (SD) time to complete the study was 22.3 (8.9) minutes. Most patients required no assistance in completing the instruments.

The socio-demographic and clinical characteristics of the patients are described in Table 1. The majority of patients were females (65%) and the mean (SD) age was 58.5 (11.5) years. In total, the patient sample consisted of 66 (36%) with breast cancer, 57 (31%) with colorectal cancer, and 61 (33%) with lung cancer. Although half of the patients were reported to be in cancer stage 4, 64 (36%) had an oncologist-reported ECOG-PS score of 0 (i.e., fully active, able to carry on all pre-disease performance without restriction); no ECOG-PS score worse than 3 was reported. Most of the patients reported to being in very good (26%) and good (29%) health states. As only five patients had an oncologist-reported ECOG-PS score of 3, these individuals were combined with the adjacent group to form the ECOG-PS 2-3 group.

**Table 1:** Socio-demographic and clinical characteristics of the patients

	Frequency (%) or Mean (± SD)			
	
	All Cancers (n = 184)	Breast Cancer (n = 66)	Colorectal Cancer (n = 57)	Lung Cancer (n = 61)
Female	119 (65)	66 (100)	25 (44)	29 (48)
Age	58.5 (± 11.5)	53.2 (± 10.9)	60.1 (± 11.1)	63.0 (± 9.8)
Marital status				
Single	24 (13)	11 (17)	6 (10)	8 (13)
Married/living with a partner	120 (65)	42 (64)	41 (72)	37 (61)
Divorced/separated/widowed	37 (20)	13 (20)	10 (18)	14 (23)
Ethnicity				
Caucasian	85 (46)	32 (48)	29 (51)	24 (61)
Asian	26 (14)	11 (17)	7 (11)	10 (17)
Other ethnicity	70 (38)	22 (33)	18 (32)	28 (46)
Employment				
Full time	55 (30)	26 (39)	20 (35)	10 (16)
Retired	66 (36)	15 (23)	32 (52)	20 (35)
Unemployed	42 (23)	17 (26)	13 (23)	18 (11)
Education				
Primary school	51 (28)	14 (21)	20 (35)	17 (29)
Secondary school	15 (8)	3 (5)	7 (12)	7 (12)
College/University	9 (5)	45 (68)	29 (51)	31 (53)
Other	9 (5)	4 (6)	1 (2)	4 (7)
Annual income (CAD)				
<$29,999	42 (24)	12 (19)	14 (25)	16 (29)
$30,000-$59,999	55 (32)	17 (27)	15 (27)	24 (54)
$60,000-$99,999	35 (20)	15 (24)	9 (16)	10 (18)
≥ $100,000	42 (24)	18 (29)	16 (29)	7 (13)
Stage of disease				
1	15 (8)	5 (8)	2 (3)	8 (14)
2	31 (17)	26 (66)	2 (3)	3 (5)
3	46 (25)	8 (12)	18 (32)	20 (34)
4	92 (50)	27 (41)	27 (48)	35 (59)
Eastern Cooperative Oncology Group				
0	64 (34.8)	33 (50.0)	18 (31.6)	13 (21.3)
1	96 (52.2)	27 (40.9)	33 (57.9)	36 (59.0)
2	15 (8.2)	1 (1.5)	4 (7.0)	10 (16.4)
3	5 (2.7)	2 (3.0)	1 (1.8)	2 (3.3)
Self-reported general health				
Excellent	14 (7.6)	1 (1.5)	9 (15.8)	4 (6.6)
Very good	48 (26.1)	20 (30.3)	16 (28.1)	12 (19.7)
Good	54 (29.3)	21 (31.8)	20 (35.1)	13 (21.3)
Fair	36 (19.6)	11 (16.7)	7 (12.3)	18 (31.0)
Poor	22 (12.0)	8 (12.1)	4 (7.0)	10 (16.4)
Very poor	5 (2.7)	3 (4.5)	1 (1.8)	1 (1.6)

### Quality of Life Scores

Table 2 displays a summary of the QOL scores obtained from the instruments used in this study. For the generic preference-based instruments, a maximum score of 1.0 was achieved but the minimum values varied. The SF-6D and HUI-3 had interquartile ranges (IQRs) of 0.14 and 0.17, respectively, which is lower than those of the EQ-5D (IQR = 0.22) and the HUI-2 (IQR = 0.31). The mean (SD) values between the two cancer-specific instruments differed; such that patients valued their QOL higher using the FACT-G (81.61 (14.14)) when compared to the QLQ-C30 (68.90 (20.36)). Seventeen (9%) patients had a best possible score for the global health status score of the QLQ-C30; none provided the best possible score for the FACT-G. Fourteen of these participants gave a score of greater than 0.95 for the HUI-2 and HUI-3; 11 gave the best possible scores for the EQ-5D, and the SF-6D.

**Table 2:** Quality of life scores of the instruments

Instrument	Mean	SD*	Median	IQR*	**Min**.	**Max**.
QLQ-C30	68.90	20.36	66.67	25.00	0.00	100.00
FACT-G	81.61	14.14	83.92	18.83	40.00	107.00
EQ-5D	0.83	0.14	0.83	0.22	0.11	1.00
SF-6D	0.73	0.11	0.74	0.14	0.44	1.00
HUI-2	0.76	0.23	0.84	0.31	-0.04	1.00
HUI-3	0.83	0.13	0.88	0.17	0.30	1.00

* SD: standard deviation; IQR: interquartile range.

Paired t-tests indicated no significant differences in mean scores of the generic preference-based instruments between females and males; married and not married; and Caucasian and non-Caucasian (results not presented). Mean EQ-5D and HUI-2 scores were found to be higher for more educated participants. We found no significant differences in mean values of the cancer-specific QOL scores when stratified by sex and age.

### Internal Consistency and Convergent Validity

Cronbach's α coefficients for the QLQ-C30 and FACT-G scales are shown in Table 3. Both instruments met the minimum standard for reliability (α = 0.70). In general, correlations between the QLQ-C30 and the FACT-G were high when scales and sub-scales were related to the same QOL domain and low when they related to different domains (Table 4). A high correlation was observed between FACT-G physical well-being and the role function (r = 0.64) and physical function scale (r = 0.55) of QLQ-C30. The functional well-being of the FACT-G was highly correlated with the role functioning (r = 0.61) and the physical functioning (r = 0.58) of the QLQ-C30. The social domains of QLQ-C30 and FACT-G were poorly correlated (r = 0.13), but the emotional subscales were strongly correlated (r = 0.76). The FACT-G global score was highly correlated with all QLQ-C30 domains, with the exception of cognitive functioning.

**Table 3:** Internal consistency and ceiling-floor effects for the EORTC QLQ-C30 and FACT-G

	Scores mean (SD)	**α**^**1**^	**α**^**2**^
**EORTC-QLQ-C30**			
Physical functioning	77.53(19.49)	0.78	0.77
Social functioning	72.12(26.23)	0.77	0.77
Emotional functioning	78.43(21.12)	0.81	0.81
Cognitive functioning	80.56(21.90)	0.82	0.81
Role functioning	72.83(26.35)	0.76	0.76
Global health status	68.75(20.46)	0.78	0.78
**FACT-G subscale**			
Physical Well-Being	21.38(5.11)	0.79	0.82
Social/Family Well-Being	23.13(4.09)	0.82	0.69
Emotional Well-Being	18.38(4.36)	0.87	0.74
Functional Well-Being	18.65(5.54)	0.83	0.80
Global health status	81.50(14.22)	0.71	0.89

Notes: ^1^α = Cronbach's alpha coefficient.

^2^α = Cronbach's alpha coefficient original version of QLQ-C30 by Aaronson and colleagues [4] and of FACT-G by Cella and colleagues [5].

**Table 4:** Pearson Correlations between the QLQ-C30 and FACT-G sub-scales

	**QLQ-C30**	**PF**	**RF**	**EF**	**CF**	**SF**
	
FACT-G	0.629	0.522	0.598	0.658	0.394	0.542
PWB	0.553	0.551	0.637	0.504	0.443	0.545
SWB	0.193^†^	0.128^†^	0.073^†^	0.187^†^	0.164^†^	0.130^†^
EWB	0.335	0.188^†^	0.343	0.761	0.201^†^	0.315
FWB	0.686	0.579	0.607	0.460	0.321	0.524

Notes: QLQ-C30 = EORTC-QLQ-C30 global score; PF = Physical functioning; RF = Role functioning;

EF = Emotional functioning; CF = Cognitive functioning; SF = Social functioning.

FACT-G = FACT-G global score; PWB = Physical well-being; SWB = Social well-being;

EWB = Emotional well being; FWB = Functional well-being

All correlations are significant at 0.05 level after Bonferroni corrections applied, except for ^†^

The correlation between the cancer-specific and the generic preference-based instruments was positive and, in general, moderate (Table 5); stronger correlations were observed between the FACT-G and the HUI-2 (r = 0.64) and HUI-3 (r = 0.61). The QOL scores from the three generic instruments moderately to strongly correlated with each other (r = 0.38-0.70).

**Table 5:** Pearson correlations for the quality of life scores for all instruments

	**QLQ-C30**	**FACT-G**	**EQ-5D**	**SF-6D**	**HUI-2**	**HUI-3**
	
QLQ-C30	1.00					
FACT-G	0.59	1.00				
EQ-5D	0.43	0.50	1.00			
SF-6D	0.48	0.47	0.62	1.00		
HUI-2	0.41	0.64	0.48	0.38	1.00	
HUI-3	0.44	0.61	0.68	0.51	0.70	1.00
EQ-VAS	0.73	0.51	0.43	0.45	0.40	0.44

All correlations are significant at 0.05 level after Bonferroni corrections applied.

### Discriminant Validity and Effect Size

Table 6 illustrates the relationships between the QOL scores and all investigated measures of cancer severity. In general, the relationships between QOL and disease severity demonstrated a monotonic gradient, such that a lower QOL was associated with greater disease severity (i.e., higher cancer stage and ECOG-PS score, and poorer self-reported health status). This expressed the ability of the instruments to discriminate between different levels of cancer severity, thereby supporting validity for all instruments for this specific population. The results revealed that there is an absence of a linear gradient with the generic preference-based measures when stratified by the patient's cancer stage; this was supported by the ANOVA results.

**Table 6:** Relationship between cancer severity variables and the QOL scores

	Mean score (SD)					
	
	QLQ-C30	FACT-G	EQ-5D	SF-6D	HUI-2	HUI-3
**Stage of cancer**						
1	73.89 (12.94) *	87.71 (12.59) *	0.84 (0.13)	0.74 (0.08) *	0.84 (0.14) *	0.81 (0.21) *
2	74.72 (16.74) *	84.31 (13.54) *	0.84 (0.15)	0.73 (0.10) *	0.88 (0.10) *	0.79 (0.23) *
3	70.64 (22.27) *	82.63 (11.87) *	0.85 (0.14)	0.76 (0.11) *	0.86 (0.11) *	0.80 (0.22) *
4	65.02 (21.16) *	78.98 (15.37) *	0.82 (0.14)	0.71 (0.11) *	0.81 (0.15) *	0.71 (0.23) *
						
**ECOG-PS**^1^						
0	76.46 (17.93)*	84.93 (13.97)*	0.83 (0.21)*	0.77 (0.11)*	0.87 (0.15)*	0.83 (0.19)*
1	67.81 (20.47)*	81.11 (14.55)*	0.78 (0.15)*	0.72 (0.08)*	0.80 (0.19)*	0.75 (0.21)*
2-3	52.08 (15.97)*	71.50 (12.13)*	0.70 (0.18)*	0.71 (0.10)*	0.76 (0.14)*	0.61 (0.24)*
						
**Self-reported health status**						
Excellent - very good	80.46 (17.41)*	88.33 (9.89)*	0.88 (0.14)*	0.78 (0.09)*	0.89 (0.09)*	0.84 (0.20)*
Good - fair	67.79 (15.03)*	81.78 (13.23)*	0.83 (0.13)*	0.72 (0.09)*	0.84 (0.13)*	0.77 (0.22)*
Poor - very poor	46.47 (19.74)*	65.78 (13.04)*	0.71 (0.11)*	0.61 (0.06)*	0.71 (0.14)*	0.54 (0.22)*

* Comparison of mean values (using ANOVA), P < 0.05.

^1^Eastern Cooperative Oncology Group performance status

Table 7 shows the effects of the cancer severity variables used in this study. Effect sizes calculated from the two cancer-specific instruments exceeded Cohen's low limits of 0.2. The QLQ-C30 (*d *= 0.40) and the FACT-G (*d *= 0.49) were generally better able to discriminate among the patients with early and late stage disease as indicated by the larger effect sizes. However, amongst the generic preference-based instruments, the HUI-2 (*d *= 0.36) and the HUI-3 (*d *= 0.24) performed better than the EQ-5D (*d *= 0.06) and the SF-6 (*d *= 0.10). Similar trends were observed for the ECOG-PS score and patient self-reported health status.

**Table 7:** Effect sizes of the cancer severity variables

	Mean score (SD)					
	
	QLQ-C30	FACT-G	EQ-5D	SF-6D	HUI-2	HUI-3
**Stage of cancer**						
1-2	74.44 (15.43)	85.47 (13.18)	0.84 (0.14)	0.73 (0.09)	0.87 (0.12)	0.80 (0.22)
3-4	66.85 (21.61)	80.20 (14.36)	0.83 (0.14)	0.72 (0.11)	0.82 (0.14)	0.74 (0.23)
**Effect size**	0.49*	0.40*	0.06*	0.10*	0.36*	0.24*
						
**ECOG-PS score^1^**						
0	76.43 (17.79)	86.12 (11.24)	0.86 (0.16)	0.77 (0.11)	0.88 (0.09)	0.83 (0.19)
1-3	64.90 (20.70)	79.27 (14.83)	0.81 (0.11)	0.71 (0.10)	0.81 (0.15)	0.72 (0.22)
**Effect size**	0.65*	0.61*	0.31*	0.57*	0.50*	0.57*
						
**Self-reported health status**						
Excellent-good	76.92 (16.06)	86.45 (10.93)	0.87 (0.13)	0.76 (0.09)	0.88 (0.11)	0.83 (0.19)
Fair-very poor	54.57 (17.83)	73.01 (15.05)	0.75 (0.12)	0.66 (0.09)	0.75 (0.14)	0.63 (0.23)
**Effect size**	**1.39**	**1.23**	**0.90**	**1.13**	**1.18**	**1.04**

* Comparison of mean values (using ANOVA), *p *< 0.05.

^1^Eastern Cooperative Oncology Group performance status

## Discussion

The key finding of this study is that the global scores of the QLQ-C30 and the FACT-G and the mean utility scores from the EQ-5D, SF-6D, and HUI2/3 are able to distinguish between cancer severity measures, namely the stage of cancer and ECOG-PS scores. The QLQ-C30 and FACT-G appear to perform better than the generic preference-based measures, as indicated by higher effect size coefficients. The EQ-5D performed less favourably than the SF-6D and HUI2/3 in discriminating patients between the cancer severity measures used in this study. This result confirms what previous studies have found regarding the unresponsiveness nature of the EQ-5D when compared with other disease-specific instruments [3,29-32]; this may be a result of the instrument having only three levels to define each item and only five items. Notably, in the field of cancer many patients report having low energy and vitality. The EQ-5D does not include an item for energy or vitality.

The comparison with the QLQ-C30 needs to be interpreted with care as an overall summary score was not obtained for this instrument. Instead, the comparison was made using the two items asking patients to rate their overall health and overall QOL during the past week (items 29 and 30). It is possible that patients may not have considered all aspects that contribute to their QOL when providing a rating for these items; thereby resulting in an inaccurate estimate. Inter-domain correlations for the two cancer-specific instruments (e.g., between physical and emotional domains) were strong. However, the correlation between the social domains of the two cancer-specific instruments was weak. The weak correlation between these domains indicated that the scales tend to measure different aspects of social problems that cancer patients face. The FACT-G social domain is primarily concerned with aspects of social life whereas the social functioning scale of the QLQ-C30 is designed to address important limitations in family and social life caused by physical complaints [4,17,33]. Such a difference, as replicated by results of this study, indicated that these two QOL instruments are designed to measure different aspect of QOL and therefore may not interchangeable.

The main advantage of using cancer-specific instruments is their items are more appropriate to the condition under investigation, unlike generic preference-based instruments, which incorporate broad domains covering all aspects of QOL. Furthermore, most items in the investigated instruments, except those in the HUI2/3, incorporate aspects of coping and adaptation. These items address the fact that patients may gradually learn to cope and adapt to their limitations in a number of ways such that, over time, the perception of the impact of their disease may be reduced. Previous studies have shown that cancer patients' emotional and functional well-being increase in the absence of corresponding increase in physical well-being, suggesting adaptation to physical limitations [34-36]. This process will have an impact on their overall QOL.

In addition to the description of the items, the valuation methods and the psychometric properties of the generic instruments may provide another explanation for the differences observed between the instruments. The SF-6D and the HUI2/3 use the SG technique for valuation, while the EQ-5D uses the TTO approach. The HUI2/3 uses multi-attribute utility (MAU) theory and multiplicative scoring models, while the other instruments use additive scoring methods. Although the scoring function for the HUI2/3 is derived from Canadian general public, the EQ-5D and SF-6D are based on a non-Canadian population. Furthermore, the scoring functions of the MAU preference-based instruments were derived from responses of the general public. As a result, this raises concerns as to whether the scoring functions of the EQ-5D and SF-6D best reflect the preferences of Canadian cancer patients, especially considering the fact that members of the general public do not often include aspects of adaptation into their valuations.

The responsiveness of the instruments needs to be evaluated longitudinally; this was difficult to evaluate due to the cross-sectional nature of the study. If a treatment strategy results in a minimum clinically important difference, the instruments will need to be able to detect this change. The most important question is whether these instruments are sensitive to changes in QOL; this can only be assessed in a longitudinal study. However, this study does investigate whether the QOL scores are correlated with cancer stage and ECOG-PS score. This is one of the strongest parts of this study given that the individual performance of the QOL instruments has been assessed previously. Results for the stage of cancer, however should be interpreted with caution due to the small size of patients for stage one (N = 15) and the issue of adaptation. In this study, we do not have information on time since diagnosis. This may influence the patients to adapt to different health states. We believe, however, the self-reported measures of general health would capture some of the adaptation effect.

The evaluative nature of these instruments also needs to be assessed, as it would be beneficial not only to measure improvements in QOL with cancer treatments but also to compare these QOL scores with those obtained for other conditions over the longer term. There is also a need to examine the measurement properties of these instruments in patients with different cancer tumour sites and in different settings. While the patients in the study were attending an outpatient visit at the cancer centre, we did not have access to information as to the type of treatment they were receiving at the time of completing the questionnaire. As such, assessing the differences in QOL between, for example, chemotherapy and radiotherapy patients could not be examined. We recognize this as a limitation, and hope to gather this information in a subsequent study.

As health is a function of both quality and length of life, the QALY is used to measure health outcomes in economic evaluation to compare the efficiency of different programs or treatment strategies in the health care system. For utilities to be of value, the scores obtained from these generic instruments need to be incorporated into a QALY measure of resource allocation decision-making. However, conducting a cost-utility analysis using the QOL values obtained in this study, only small changes will be observed when using generic instruments especially when comparing treatments for different cancer stages. Combined with the poor sensitivity to detect subtle changes in QOL, these results indicate that generic preference-based instruments may not be appropriate for comparing cancer treatments. As such, a cancer-specific preference-based measure would need to be developed to overcome the limitations of using generic instruments. A measure such as this would ensure that the utilities used in economic evaluation better reflect the impact of the health condition under investigation [29,37-40]. This is achieved by developing an algorithm to map between the cancer-specific and generic preference-based instruments; results from such a study are beyond the scope of the current work and will be presented in a future paper.

In conclusion, cancer-specific and generic preference-based instruments were demonstrated to be valid in discriminating across levels of ECOG-PS scores and self-reported health status. However, the usefulness of the generic instruments may be limited if they are not able to detect small changes in health status within cancer patients. This raises concerns regarding the appropriateness of these instruments when comparing different cancer treatments within an economic evaluation framework. The results demonstrate that the SF-6D and HUI2/3 appear to be better at discriminating patients between different severities of disease than the EQ-5D.

Researchers and practitioners should be mindful that some instruments may have greater 'sensitivity' to capturing QOL experiences in cancer patients. Administering both cancer-specific and generic preference-based measures in clinical trials will still allow valuable information to be gained. The simultaneous use of both types of instruments would allow researchers to develop a statistical algorithm to map between the cancer-specific and generic preference-based instruments; results from such a study will be presented in a future paper. Given the importance relevance of this research topic, further work is merited.

## List of Abbreviations

ANOVA: Analysis of Variance; EORTC QLQ-C30: European Organization for Research and Treatment of Cancer Quality of Life Questionnaire Core 30; EQ-5D: EuroQol 5D; FACT-G: Functional Assessment of Cancer Therapy - General; QOL: Quality of Life; HUI 2: Health Utilities Index Mark 2; HUI 3: Health Utilities Index Mark 3; QALY: Quality-Adjusted Life Years; SF-6D: Short Form 36 Health Survey.

## Competing interests

The authors declare that they have no competing interests.

## Authors' contributions

PT conceived and designed the study, oversaw all stages of data collection and entry, done the analysis, and drafted the manuscript. SP gave feedback on design and analysis. SP, HM, KvH, SC, BM, and KG reviewed the manuscript. SC, BM and KG assisted with recruitment of patients. KvH supervised data collection. HM assisted in editing the draft manuscript. All authors read and approved the final manuscript.
